# Validation of an 8-item Recovery Assessment Scale (RAS-8) for people with schizophrenia in China

**DOI:** 10.1186/s12955-021-01763-3

**Published:** 2021-04-13

**Authors:** Si-jia He, Yan-wen Fang, Zi-xin Huang, Yu Yu

**Affiliations:** 1 Department of Sociology, School of Public Management, Central South University, Lushan South Road 932, Changsha, 410083 Hunan China; 2 Department of Public Health, Faculty of Medicine, University of Queensland, 20 Weightman St, Herston, QLD 4006 Australia; 3 Department of Social Medicine and Health Management, Xiangya School of Public Health, Upper Mayuanlin Road 238, Changsha, Hunan 410008 China; 4 Division of Prevention and Community Research, Yale School of Medicine, 389 Whitney Avenue, New Haven, CT 06511 USA

**Keywords:** Recovery Assessment Scale (RAS), Short form, Psychometric testing, Reliability, Validity, Confirmatory factor analysis, Schizophrenia, Chinese

## Abstract

**Background:**

The 24-item Recovery Assessment Scale (RAS) is the most widely-used and well-validated tool for measuring recovery for people with mental illness. The current study aims to assess the reliability and validity of an 8-item short form of RAS (RAS-8) among a Chinese sample of people living with schizophrenia.

**Methods:**

A sample of 400 people living with schizophrenia were recruited for scale validation. Internal consistency was tested by calculating Cronbach's α. Test–retest reliability was calculated using the intraclass correlation coefficient (ICC) for the total score and weighted kappa for each item. Factor structure was tested with confirmatory factor analysis, and concurrent validity was examined by investigating the correlation of the RAS-8 with patient symptoms, disability, depression, anxiety, patient functioning, quality of life and general health.

**Results:**

The RAS-8 full scale and subscales showed good internal consistency with Cronbach’s alpha ranging from 0.87 to 0.92. ICC of 0.99 and weighted kappa ranged from 0.62 to 0.88, which generally indicates good test–retest reliability. The findings supported an a priori two-factor structure, χ^2^/df = 2.93, CFI = 0.98, TLI = 0.98, RMSEA = 0.07, SRMR = 0.035. Concurrent validity of the RAS-8 was further supported by its significant negative correlations with patient symptoms (r =  −0.24, p < 0.01), disability (r =  −0.30, p < 0.01), depression (r =  −0.16, p < 0.05), and anxiety (r =  −0.14, p < 0.05), and its significant positive relationships with patient functioning (r = 0.26, p < 0.01), quality of life (r = 0.39, p < 0.01) and general health (r = 0.34, p < 0.01).

**Conclusions:**

This study confirmed the reliability and validity of an 8-item short-form RAS for people living with schizophrenia in Chinese communities. The validation of the RAS-8 allows for its use as an alternative for the full RAS as a rapid assessment tool in clinical and research settings. The findings are discussed for their implications for application and validation with other populations and in other countries.

## Background

Recovery in mental health is an ongoing holistic process that can be defined as ‘a way of living a satisfying, hopeful and contributing life even with limitations caused by mental illness’ [1]. The past few decades have seen a significant transformation from a disease-oriented system of mental health care focused on symptom reduction and functional improvement to a person-centered recovery system that emphasizes the self-directed pursuit of a personally meaningful life despite the effects of mental illness [1–3]. Recovery is conceptualized as both a process and an outcome, which is reflected not only in improvement in the disorder itself (resolution), but also an adjustment to the disorder (readjustment) and an adaptation to living with the disorder (redefinition) [4]. Among the key features of recovery are hope and optimism about the future, involvement in meaningful activities, a positive identity, control of one’s life, supportive relationships, connectedness to others, self-empowerment, and reduced feelings of stigma [5–8]. This concept of recovery has become the driving philosophy underlying the development of international, national, and local mental health policy and services for people in recovery of mental illness, and it is likely to remain a guiding influence for decades to come [9].

The growing development and implementation of evidence-based, recovery-oriented mental health services necessitates a valid and reliable recovery assessment tool to evaluate the recovery orientation of mental health care and the outcomes of recovery-oriented services [9–12]. Over the years, a number of self-completed questionnaires on personal recovery have been developed by practitioners and researchers around the world and summarized in narrative reviews [13–16]. Although no gold-standard measure of recovery has yet been proposed, studies around the world consistently endorsed the Recovery Assessment Scale (RAS) [17] as the pioneering and most widely used scale globally. The RAS was originally developed based on a qualitative analysis of recovery narratives among people in recovery and included 41 self-reported items that measure subjective perceptions [17, 18]. A further study involving 1824 people with serious mental health illness resulted in a final version of 24 items in five subscales [19] supported by factor analysis and consistent with earlier conceptual research on recovery [1–3]: (a) Personal confidence and hope (items 7, 8, 9, 10, 11, 12, 13, 14 and 21), (b) Willingness to ask for help (items 18, 19 and 20), (c) Goal and success orientation (items 1, 2, 3, 4 and 5), (d) Reliance on others (items 6, 22, 23 and 24), and (e) No domination by symptoms (items 15, 16 and 17).

Ever since its development, the RAS has been widely used in both English and non-English speaking countries [20, 21], applied in hospital and community-based services [22], and endorsed by researchers and consumers [13–16]. For instance, in the review of 11 instruments on recovery, Burgess et al. [13] found the RAS met all the criteria for a quality assessment instrument of recovery practice due to its concept relevance, administration ease, appropriate development and validation process, consumer perspective, applicability and acceptability. Law et al. [15] conducted a user-informed review and also suggested the RAS as the most acceptable and valid measure currently available. In a further review of studies on RAS psychometric properties by Salzer and Brusilovskiy [9], the RAS showed good reliability including internal consistency and test–retest/inter-rater reliability, sensitivity to change over time, as well as consistent means and factor structures across studies. Furthermore, the RAS also showed good validity by its positive and significant correlations with hope, self-esteem, empowerment, quality of life, and other recovery-based measures; as well as negative and significant correlations with symptoms, disability and psychological distress [17, 19, 23]. Based on these empirical study results, the RAS has been recommended as a measure of recovery for both clinical evaluations and research [13, 14].

Although the 24-item RAS has been the most widely-used and well-validated tool for recovery measurement, a review showed that other item numbers have also been used including 20, 22, 24, 41, 42 and 50 [9]. Another cross-sectional multi-center validation study of the 24-item RAS conducted in a Norwegian context found some item redundancy among the factors (such as Personal confidence and hope, Goal and success orientation and Not dominated by symptoms) [24]. This finding is further echoed in a recent study examining the factor structure of the 24-item RAS in a German sample, which supported a 14-item RAS by confirmatory factor analysis [23]. All these results suggest a revisit of item categorization under the original theory, as well as item selection for a brief version. A short form measurement scale is promising in both clinical and research settings for ease of administration, minimization of respondent burden, and quick screening in busy clinical settings [25].

In the current study, we proposed an 8-item RAS extracted from two domains of the 24-item RAS: “goal and success orientation” (items 1, 2, 3, 4 and 5), and “no domination by symptoms” (items 15, 16 and 17). These 8 items were chosen because they cover both an illness-focused recovery (no domination by symptoms) orientation and a consumer/person-centered recovery (goal and success orientation) orientation [1–3]. Consistent with these orientations, these items emphasize purpose and empowerment as central components of recovery [6, 19, 26]. The current study seeks to validate an 8-item short form of the RAS in a community sample of people with schizophrenia in China.

## Methods

### Participants and procedure

Data were obtained from a baseline assessment of a larger research project focused on adaptation among people with schizophrenia and their families in China that was funded with a National Natural Science Foundation grant in China.

The study sample consisted of 400 people with schizophrenia (50% male, n = 200), with a mean age of 46.87 years (SD = 10.99, range = 18–77). The participants were recruited from 12 community mental health centers through the “686 Program”, which is China's largest demonstration project aimed at integrating hospital and community services for serious mental illness [27]. Inclusion criteria includes: 1) being registered in the “686 Program”; 2) fulfilling the Chinese Classification of Mental Disorders-3 (CCMD-3) or the International Classification of Diseases-10 (ICD-10) criteria for schizophrenia; 3) living with at least one family member; and 4) being ≥ 18 years of age; 5) able to read and communicate. They were excluded if they were: 1) not registered in the “686 Program”; 2) with diagnosis other than schizophrenia; 3) living alone; 4) younger than 18 years of age; 5) being too mentally disabled or too illiterate to read or communicate. Most of the participants were unemployed (89.5%, n = 358) and with an education level of middle and high school (67.8%, n = 271). The mean duration of schizophrenia was 21.42 years (SD = 10.62). The largest proportion of participants were married or living with partners (43%, n = 172), followed by single (37.5%, n = 150), a pattern consistent with those reported in other studies among PLS in China [28, 29]. Table 1 shows the participants’ demographic information. Medication adherence was generally good, with 90.3% taking medication every day based on the doctor’s advice (full medication adherence), a rate that is comparable to those reported in other studies on PLS registered into the 686 Program [30].

**Table 1:** Demographic characteristics of the participants (N = 400)

Variables	M (sd)/N (%)
Age	46.87 (10.99)
*Gender*	
Male	200 (50%)
Female	200 (50%)
*Employment*	
Employed	42 (10.50)
Unemployed	358 (89.50)
*Education*	
Primary and below	75 (18.75)
Middle and high	271 (67.75)
College and above	54 (13.50)
*Marrital Status*	
Single	150 (37.50)
Married/cohabited	172 (43.00)
Else(divorced/separated/widowed)	78 (19.50)
Illness duration	21.42 (10.62)
*Full medication adherence*	
No	28 (7.00)
Yes	361 (90.25)

The study was approved by the Institutional Review Board of the Xiangya School of Public Health of Central South University (No.: XYGW-2019-029). A random sample of participants was recruited from 12 community mental health centers through the 686 Program that distribute free anti-psychotic medicines to registered clients monthly. Eligible participants were identified by medicine distribution staff and invited to participate in the study. After providing written informed consent, participants received face-to-face interviews by field psychiatrists, who also made clinical assessment of each participant and filled in the study questionnaire. Furthermore, we randomly selected 25 participants to complete received the same RAS-8 one month later to examine test–retest reliability.

### Measurements

#### 8-item Recovery Assessment Scale (RAS-8)

The RAS-8 was used to evaluate the recovery process among people in recovery of mental illness; it consisted of two domains: goal and success orientation (items 1, 2, 3, 4 and 5), and no domination by symptoms (items 6, 7 and 8). Sample items of the RAS include ‘‘I have goals in life that I want to reach’’ and ‘‘My symptoms interfere less and less with my life’’. Items are rated on a 5-point scale from 1 (strongly disagree) to 5 (strongly agree), with higher total scores indicating better perceived recovery.

#### 18-item Brief Psychiatric Rating Scale (BPRS-18)

The 18-item Brief Psychiatric Rating Scale (BPRS-18) was used to assess symptomatology across a comprehensive set of common symptom characteristics in psychiatric patients [31]. It covers five domains of clinical symptoms: affect, positive symptoms, negative symptoms, resistance, and activation as proposed by Shafer [32]. Items are rated on an 8-point scale from 0- “not assessed”, 1- “not at all” to 7- “extremely severe” by a clinician to assess symptom severity. The total score ranges from 0 to 126, with higher score representing greater severity of symptoms. The BPRS-18 has been frequently used in schizophrenia with well-established psychometric properties [31, 32]. In the current study, the BPRS-18 showed good internal consistency, with a Cronbach’s alpha of 0.85.

#### 12-item World Health Organization Disability Assessment Schedule 2.0 (WHODAS 2.0-12)

The 12-item World Health Organization Disability Assessment Schedule 2.0 (WHODAS 2.0-12) [33] is a standard measure of disability and functional impairment promoted by the World Health Organization. It covers six domains of function: cognition, mobility, self-care, getting along with people, life activities, and participation in society [33]. Items are rated on a 5-point scale from 0- “no difficulty” to 4- “extreme difficulty” to assess the level of difficulty experienced while performing the activities. The total score ranges from 0 to 48, with higher score indicating higher disability. The Chinese version of WHODAS 2.0-12 also showed good psychometric performance [34, 35]. In the current study, the WHODAS 2.0-12 showed good internal consistency, with a Cronbach’s alpha of 0.89.

#### Patient Health Questionnaire-9 (PHQ-9)

Patient Health Questionnaire-9 (PHQ-9) [36] was used to screen for depression by asking whether respondents have experienced various symptoms in the past two weeks, such as: losing interest in doing things, feeling down or depressed, having difficulty with sleeping, and thoughts of suicide. Items are rated on a 4-point scale from 0 = "not at all" to 3 = "nearly every day" to assess the severity degree of depression symptoms. The total score ranges from 0 to 27, with higher score indicating more depressive symptoms. The Chinese version of PHQ-9 has also been widely proven to be both culturally acceptable and psychometrically valid in various parts of China [37–41]. In the current study, the PHQ-9 showed good internal consistency, with a Cronbach’s alpha of 0.92.

#### Generalized Anxiety Disorder Scale-7 (GAD-7)

The Generalized Anxiety Disorder Scale-7 (GAD-7) [42] was used to screen for anxiety by asking whether respondents have experienced symptoms in the past two weeks, such as: feeling nervous, having trouble relaxing, and feeling afraid. Each item is rated on a 4-point scale from 0 = "not at all" to 3 = "nearly every day" to assess the severity degree of anxiety symptoms. The total score ranges from 0 to 27, with higher score indicating more anxiety symptoms. The Chinese version of GAD-7 has also been widely proven to be both culturally acceptable and psychometrically valid in various parts of China [43–46]. In the current study, the GAD-7 showed good internal consistency, with a Cronbach’s alpha of 0.96.

#### Global Assessment of Functioning (GAF)

The Global Assessment of Functioning (GAF) was used to measure a person’s psychological, social, and occupational functioning on a hypothetical continuum of mental health-illness ranging from 1 to 100 [47], with higher score indicating better functioning. Examples are provided for each ten-level interval. The GAF has also been widely used in clinical assessment with satisfactory psychometric properties established [48, 49]. In the current study we assessed the functional level of people with schizophrenia over the past 1 month.

#### World Health Organization Quality of Life Brief Scale (WHOQOL-BREF)

The World Health Organization Quality of Life Brief Scale (WHOQOL-BREF) [50] is a generic cross-cultural instrument to measure quality of life and is available in more than 40 countries [51]. Here we only used the first 2 questions to measure overall quality of life and general well-being. Quality of life was assessed by asking “How do you evaluate your quality of life in the past two weeks?” on a 5-point scale from 1 = “very bad” to 5 = “very good”. General well-being was assessed by asking “Are you satisfied with your health status?” on a 5-point scale from 1 = “very unsatisfied” to 5 = “very satisfied”.

### Statistical analyses

Sociodemographic characteristics of the sample were examined using descriptive statistics including mean and standard deviation for continuous variables and frequencies for categorical. Internal consistency was tested by calculating Cronbach's α, with a recommended level of 0.70 or above indicating good internal consistency based on the criterion by Nunnally [52]. Test–retest reliability was calculated in a subsample of these participants (n = 25) who were surveyed again 1–2 weeks later to calculate the intraclass correlation coefficient (ICC) for the total score and kappa for each item. A recommended ICC of 0.70 or above and a kappa of 0.60 and above indicates good test–retest reliability [53].

Factorial validity was evaluated by confirmatory factor analysis (CFA) to test the a priori two-factor structure using structural equation modeling (SEM). Model fit was assessed using a combination of fit indices including relative Chi-square (χ^2^/df), comparative fit index (CFI), Tucker–Lewis Index (TLI), root mean square error of approximation (RMSEA), and standardized root mean square residual (SRMR). Relative chi-square is the ratio of chi-square to degrees of freedom, with a recommended level of < 3 for acceptable model fit [54]. Values for CFI and TLI range between 0 and 1, with values closer to 1 or > 0.90 indicative of data fitness [55]. An RMSEA ranging from 0.08 to 0.10 shows a moderate fit and below 0.08 indicates a good fit [56, 57]. The acceptable value for SRMR is < 0.10, with values < 0.08 indicating adequate fit, and values below 0.05 indicating good fit [56, 57].

Concurrent validity of the RAS-8 was tested using Spearman’s Rank Correlations with expected significant negative correlations with client symptoms (as measured by BPRS-18), depression (as measured by PHQ-9) and anxiety (as measured by GAD-7), as well as an expected significant positive correlation with client functioning (as measured by GAF), quality of life, and well-being (as measured by the first two questions of the WHOQOL-BREF-2). In addition, Cohen’s [58] guidelines were used to determine the strengths of the correlation coefficients between RAS and other measures, with r around 0.1 indicating small effect sizes, r around 0.3 indicating medium effect sizes, and r > 0.5 indicating large effect sizes. All data were analyzed using STATA version 16. Values of p less than 0.05 were considered statistically significant (two-tailed test).

## Results

### Internal consistency reliability

Cronbach’s alpha coefficient was 0.91 for the total score of the RAS-8, 0.92 for the subscale of goal and success orientation, and 0.87 for the subscale of no domination by symptoms. All these results indicate good internal consistency reliability.

### Test–retest reliability

The ICC for the total score was 0.99 (p < 0.001), exceeding the recommended standard of 0.70. Kappa for each item was also examined. All items showed good reliability with kappa higher than the recommend 0.60 (ranging from 0.62 for item 8 to 0.88 for item 2).

### Factorial validity

A CFA was conducted to test the a priori two-factor structure of the RAS-8. The relative Chi-squares (χ^2^/df = 2.93) was lower than 3, indicating the fitness of the model [54]. The values of CFI and TLI were both being 0.98 and close to 1, also showing goodness-of-fit for the data [55]. A RMSEA value of lower than 0.08 (0.07) and a SRMR value of lower than 0.04 (0.035) further supported a good fit [56, 57]. In sum, all the fit indices revealed a good model fit for the two-factor RAS-8. Table 2 displays the means, standard deviations, and factor loadings of all 8 items. All items loaded well in their respective domains, with factoring loading ranging from 0.72 to 0.93 for subscale of goal and success orientation, and 0.74–0.89 for the subscale of no domination by symptoms. Table 3 shows a correlation of 0.59 between the two subscales of goal and success orientation and no domination by symptoms, indicating a large effect size.

**Table 2:** Descriptive statistics and factor loadings of all 8 items of the Recovery Assessment Scale (RAS-8)

Items	Mean(SD)	Factor loading
Factor 1: Goal and success orientation (α = 0.92)		
1. I have a desire to succeed	2.86 (1.73)	0.72
2. I have my own plan for how to stay or become well	2.41 (1.38)	0.80
3. I have goals in life that I want to reach	2.47 (1.47)	0.93
4. I believe I can meet my current personal goals	2.30 (1.39)	0.88
5. I have a purpose in life	2.49 (1.47)	0.88
Factor 2: No domination by symptoms (α = .87)		
6. Coping with my mental illness is no longer the main focus of my life	2.54 (1.44)	0.74
7. My symptoms interfere less and less with my life	2.65 (1.46)	0.89
8. My symptoms seem to be a problem for shorter periods of time each time they occur	2.58 (1.45)	0.87
Correlation between Factor 1 and Factor 2	0.60

**Table 3:** Correlations of RAS-8 and its two subscales with other variables

Variables	1	2	3	4	5	6	7	8	9	10
1. RAS-8	1.00									
2. Goal and success orientation	0.93**	1.00								
3. No domination by symptoms	0.83**	0.59**	1.00							
4. Symptoms(BPRS-18)	− 0.24**	− 0.18**	− 0.27**	1.00						
5. Disability (WHODAS 2.0-12)	− 0.30**	− 0.26**	− 0.31**	0.43**	1.00					
6. Depression (PHQ-9)	− 0.16**	− 0.10*	− 0.22**	0.46**	0.45**	1.00				
7. Anxiety (GAD-7)	− 0.14**	− 0.08*	− 0.20**	0.47**	0.40**	0.75**	1.00			
8. Functioning (GAF)	0.26**	0.21**	0.29**	− 0.58**	− 0.43**	− 0.36**	− 0.31**	1.00		
9. Quality of life (WHOQOL-BREF-1)	0.39**	0.37**	0.36**	− 0.41**	− 0.35**	− 0.35**	− 0.36**	0.35**	1.00	
10. General Health (WHOQOL-BREF-2)	0.34**	0.27**	0.38**	− 0.45**	− 0.37**	− 0.48**	− 0.49**	0.37**	0.71**	1.00**

Spearman correlation using pairwise deletion for missing values

*RAS-8* 8-item Recovery Assessment Scale, *BPRS-18* 18-item Brief Psychiatric Rating Scale, *WHODAS 2.0-12* 12-item World Health Organization Disability Assessment Schedule 2.0, *PHQ-9* Patient Health Questionnaire-9, *GAD-7* Generalized Anxiety Disorder Scale-7, *GAF* Global Assessment of Functioning, *WHOQOL-BREF-1* The first question of the World Health Organization Quality of life brief scale, *WHOQOL-BREF-2* The second question of the World Health Organization Quality of life brief scale

^*^P < 0.05, ** P < 0.01

### Concurrent validity

Drawing on the existing literature, recovery was negatively related to patient symptoms, disability, depression and anxiety, and positively associated with patient functioning, quality of life, and well-being. The correlations between RAS-8 and BPRS-18, WHO-DAS II, PHQ-9, GAD-7, GAF and WHOQOL-BREF-2 were calculated to determine the concurrent validity of the RAS-7. As shown in Table 3, the total RAS-8 and its two subscales were all inversely and significantly related to BPRS-18 (r ranged from − 0.18 to − 0.27), WHO-DAS II (r ranged from − 0.26 to − 0.31), PHQ-9 (r ranged from − 0.10 to − 0.22), GAD-7 (r ranged from − 0.08 to − 0.20), as well as positively and significantly related to GAF(r ranged from 0.21 to 0.29) and WHOQOL-BREF-2 (r ranged from 0.36 to 0.39 for quality of life; r ranged from 0.27 to 0.38 for general health). All these correlations were of medium size effect with p values below 0.05, corroborating concurrent validity of the RAS-8.

## Discussion

The concept of person-centered recovery has gained increasing prominence over the past decade. A psychometrically sound measure of recovery is essential not only for recovery assessment but also for development of future evidence-based interventions to evaluate recovery-oriented care services [21]. Such a measure can benefit not only those reporting on their recovery experience but also their caregivers and service providers [21]. Although the Recovery Assessment Scale (RAS) [17] has been endorsed as the most widely used scale globally, a psychometrically sound short form still awaits development. The present study sought to develop an 8-item RAS short form by using two domains drawn from the original RAS–goal and success orientation and no domination by symptoms; and test its psychometric performance among a sample of Chinese people with schizophrenia. We tested internal consistency reliability, test–retest reliability, factorial validity, and concurrent validity, and our findings showed that the RAS-8 full scale and subscales were psychometrically sound. Overall, the RAS-8 showed good internal consistency and test–retest reliability, and confirmatory factor analysis supported the a priori two-factor structure with favorable model fit indices. Concurrent validity was also supported by significant negative correlations with patient symptoms, disability, depression, and anxiety, and significant positive relationships with patient functioning, quality of life and general health. Thus, the RAS-8 demonstrated psychometrically sound properties for assessing the subjective experience of recovery among people with schizophrenia.

Cronbach’s alpha coefficients for the total scale and two subscales exceeded 0.85, indicating high internal consistency reliability of the RAS-8. This finding is comparable to previous observations showing Cronbach’s alphas greater than 0.70 for the full RAS scale in both Chinese and non-Chinese samples [19–21, 24]. Although Cronbach’s alpha coefficients are sensitive to the number of items and decrease with reduced items [59], the relatively higher Cronbach’s alpha coefficients of the shorter version of RAS than the full version further demonstrates item homogeneity and internal consistency of the RAS-8. High test–retest reliability was supported by high ICC of the total score and high weighted kappa for each item, also showing the stability of RAS-8 in assessing recovery over time. However, test–retest reliability findings must be interpreted with caution because of the small sample size. Future research is warranted to examine test–retest reliability using a larger sample size.

Confirmatory Factor Analysis confirmed the a priori two-factor structure of RAS-8: goal and success orientation (items 1, 2, 3, 4 and 5) and no domination by symptoms (items 6, 7 and 8) with generally good model fit indices. This finding partially resonates with the original factor analysis of the full RAS scale that includes the two conceptually-distinguishable domains and shows robustness of the factor structure of the RAS even with shorter item numbers [19]. The two-factor structure is also in accordance with our theoretical hypothesis that the RAS-8 covers both disease-oriented recovery (no domination by symptoms) and consumer/person-centered recovery (goal and success orientation).

Concurrent validity of the RAS-8 was demonstrated by its moderate negative correlations with patient symptoms, disability, depression, and anxiety, as well as its moderate positive associations with patient functioning, quality of life and general health. Although previous studies conceptually distinguished between recovery and no psychiatric symptoms or no disability [60, 61], this distinction has now been weakened by increasing evidence showing a negative correlation between recovery and psychiatric symptoms or disability [17, 20, 21], also reflected in the current study. These findings show that symptom management and disability decrease are key components of the recovery process. The negative correlations of RAS-8 with depression and anxiety were consistent with the findings by McNaught et al. [62] showing a correlation coefficient of -0.43 between RAS and psychological distress. What is noteworthy is that RAS-8 shows strongest correlation with patient functioning, which resonates with the idea that functional recovery as measured by GFA is distinct from, but complementary to, personal recovery as measured by RAS [63, 64]. The findings were also in accordance with previous studies showing similar results [20, 21] and further supported the validity of the RAS-8. Also consistent with our expectation, the RAS-8 was positively correlated with quality of life and general health, as measured by the first two items of the WHOQOL-BREF, suggesting that those with better recovery also enjoy higher quality of life and are more satisfied with their general health. These results paralleled previous findings [19, 65] and provided additional support for the concurrent validity of the RAS-8.

Although this study presented strong empirical evidence for the reliability and validity of the RAS-8, some possible limitations should also be considered [66]. First, the generalization of the findings should be done with caution because participants were restricted to adult individuals living with schizophrenia in China. The RAS-8 should be validated by future research in other samples, such as those who are younger, with other diagnoses, and in other countries. Second, the cross-sectional descriptive design makes it more difficult to test sensitivity to change of the RAS-8. Future research should fill in this gap by conducting longitudinal studies to see whether RAS-8 is sufficiently sensitive to reflect changes after interventions and over time. Third, the relatively small sample size used in the test–retest reliability assessment is a limitation to be examined with a larger sample for retest in future research. Fourth, we didn’t include the full 24-item RAS in our analysis and thus cannot assess criterion validity. Future research may consider including the full scale to calculate the correlation between RAS-8 and the full scale. Finally, the present study did not account for other measurement properties of the RAS-8, such as measurement precision, item difficulty, and response category structure [67], which may be tested by other analyses such as Rasch analysis in future validation studies [68].

## Conclusions

This study confirmed the internal consistency, test–retest reliability, factorial validity, and concurrent validity of an 8-item RAS short form (RAS-8) among people with schizophrenia in China. The RAS-8 may be useful as a reliable and valid self-report measure of recovery. The validation of RAS-8 enables its usage as an alternative for the full RAS as a rapid assessment tool in busy clinical and research settings.

## Data Availability

The datasets used and/or analyzed during the current study are available from the corresponding author on reasonable request.

## Abbreviations

BPRS: Brief Psychiatric Rating Scale
CCMD-3: Chinese Classification of Mental Disorders-3
CFA: Confirmatory factor analysis
CFI: Comparative fit index
GAD-7: Generalized Anxiety Disorder Scale-7
GAF: Global Assessment of Functioning
ICC: Intraclass correlation coefficient
ICD-10: International Classification of Diseases-10
PHQ-9: Patient Health Questionnaire-9
RAS: Recovery Assessment Scale
RMSEA: Root mean square error of approximation
SEM: Structural equation modeling
SRMR: Standardized root mean square residual
TLI: Tucker–Lewis Index
WHODAS 2.0: World Health Organization Disability Assessment Schedule 2.0
WHOQOL-BREF: World Health Organization Quality of Life Brief Scale
